# Can Leadership Enhance Patient Satisfaction? Assessing the Role of Administrative and Medical Quality

**DOI:** 10.3390/ijerph16173212

**Published:** 2019-09-03

**Authors:** Muhammad Asif, Arif Jameel, Noman Sahito, Jinsoo Hwang, Abid Hussain, Faiza Manzoor

**Affiliations:** 1 School of Public Affairs, Zijingang Campus, Zhejiang University, Hangzhou 310058, China (M.A.) (A.J.) (A.H.); 2 Department of City & Regional Planning, Mehran University of Engineering & Technology, Jamshoro 76062, Pakistan; 3 The College of Hospitality and Tourism Management, Sejong University, 98 Gunja-Dong, Gwanjin-Gu, Seoul 143-747, Korea; 4 Department of Agricultural Economics and Management, School of Management, Zhejiang University, Hangzhou 310029, China

**Keywords:** participative leadership, administrative quality, medical quality, patient satisfaction, MBNQA, structural equation modeling

## Abstract

This paper aimed to investigate the relationships between participative leadership (PL), administrative quality (AQ), medical quality (MQ), and patient satisfaction (PS) using the Malcolm Baldrige National Quality Award Healthcare Criteria (MBNQA) criteria. The study further examined the intervening influence of administrative quality and medical quality on the relationship between participative leadership and patient satisfaction. The data was obtained from 123 public sector hospitals in Pakistan. We employed confirmatory factor analysis (CFA) and structural equation modeling (SEM) techniques to test the structural model. From the study results, we found significant and positive relationships between participative, administrative quality, medical quality, and patient satisfaction. In addition, our research found administrative quality and medical quality as potential mediators on PL-PS relation. Adopting participative leadership as an exogenous factor, and both administrative and medical quality as potential mediators of patient satisfaction, provided new insights into MBNQA criteria.

## 1. Introduction

In recent years, healthcare spending has been increasing rapidly in order to fulfill patient needs associated with proper disease diagnosis procedures, provision of adequate medication and clinical services [1]. Adopting new technology, advanced diagnostic and treatment procedures, an increase in physician and institutions’ liabilities, and using limited resources are the important features that are linked with hospital expenses [2]. To sort out this issue, healthcare institutions are shifting toward a market-oriented strategy and developing efficient quality improvement programs by adopting patient satisfaction (PS) as the benchmark of institutional performance [3,4]. Shi, et al. [5] argued that it is very crucial for healthcare organizations to opt for an appropriate measurement approach for patient satisfaction (PS) in order to provide efficient and effective clinical services.

Previous studies have provided several healthcare service quality structures that are linked to better organizational performance [6,7,8]. Dividing service quality dimension into two separate categories—medical quality (MQ) and administrative quality (AQ) is the most appropriate framework in the healthcare industry. The dimension of medical quality (MQ) is associated with technical aspects provided by the physicians and other medical staff to the patients while administrative quality (AQ) consist of non-technical aspects associated with different out sided procedures. In a broader way, medical quality deals with ‘what’ type of services that are provided to clients while administrative quality determines ‘how’ these services are provided. To our knowledge, no research has been conducted that is relevant to patient satisfaction (PS) by focusing on medical quality and administrative quality.

Prior research considered “quality-minded” leadership as an essential element of patient satisfaction, service quality [9,10], and institutional performance [11,12,13]. Similarly, researchers have recognized the significance of an effective leadership style in the healthcare system and its critical role in improving patient satisfaction [14,15]. For instance, the Malcolm Baldrige National Quality Award Healthcare Criteria (MBNQA) that was established in 1987, and then in 1995, it was recognized by healthcare organizations and described healthcare leadership as “How your organization’s senior leaders address values and performance expectations, as well as a focus on patients and other key customers and stakeholders, empowerment, innovation, learning, and organizational directions.”.

Since the last two decades, the economic growth of Pakistan has been declining due to the low level of international investment, limited resources (water and financial), poverty, lack of policy implementation, and selection of inappropriate standards for quality and productivity assessment [16]. Taking these issues into account, the National Productivity Organization (NPO) introduced a quality improvement program “Prime Minister Quality Award (PMQA)” in 2010 [17]. This PMQA program has been developed under the recommendations of MBNQA criteria which are recognized by more than 70 nations worldwide, and 17 of them are in Asia. The main purpose of this award was to recognize and implement proper quality standards in order to achieve higher performance and client satisfaction [18].

In MBNQA structural criteria, an effective leadership style is considered an important exogenous factor of achieving institutional performance and desired objectives [9,11]. Previous studies confirmed the significant impact of using leadership as an exogenous factor in healthcare management system [19,20]. Similarly, Fry, et al. [21] adopted spiritual leadership organizational performance. Since the establishment of MBNQA criteria, many healthcare institutions adopted it to improve their quality and performance associated with patient-centered care [22,23,24,25], and research has been done to evaluate the influence of participative leadership on patient satisfaction through its administrative quality and medical quality.

Therefore, we proposed a structural model to measure patient satisfaction using participative leadership as an effective leadership style. In our model, we also test the influences of medical and administrative quality. We used administrative quality and medical quality as the intervening factors with the purpose to improve patient satisfaction (PS) and validate the MBNQA criteria. We employed CFA and SEM techniques to evaluate the structural model [14,26]. The present study contributes to the healthcare management sector in different ways. First our research confirmed that participative leadership is an effective leadership style to improve patient satisfaction and administrative and medical quality in the healthcare sector. Second, the significant indirect effect of administrative quality and medical quality on PL-PS relationship provided greater insights into leadership study in healthcare. Our findings revealed that administrative quality and medical quality positively mediate the PL-PS relationship and improves patient satisfaction (PS). Third, our research empirically validated the MBNQA model in the healthcare sector using participative leadership (PL) as an exogenous factor. Finally, our research proved the MBNQA model as a successful measure of healthcare performance in a developing country.

## 2. Theory and Hypotheses

### 2.1. LeaderShip

In 1987, the MBNQA criterion was established to identify different types of qualities (e.g., policy, procedural, process, information qualities, etc.) for the betterment of the institutions (manufacturing, health, service sector, small business, education, and non-profit organizations) and to endorse these qualities practically. For this purpose, more than five-hundred administration and quality specialists established the unique MBNQA Criterion and structural model [27]. At the start, this model was restrained to production companies and services-for-profit organizations. In 1995, an experimental study was commenced to establish a particular award criterion solely to focus on the quality programs in healthcare institutions. The criterion also offered a renowned MBNQA structural model that associates leadership with different aspects like procedural management, personnel management, and growth, long term planning, information, and investigation. These five dimensions then connected to the client and shareholder’s gratification and performance outcomes [11]. Meyer [28] empirically investigated the Baldrige Healthcare model and found leadership to be the external factor of improved fiscal performance.

### 2.2. Medical Quality

Medical quality (MQ) can be described as the competency of a hospital to attain desired outcomes of patient health by means of clinical diagnosis, procedural treatment, and physically or physiologically influence on patients. Services quality is of two types: medical quality (MQ) and administrative quality (AQ) [5]. Medical quality related to technical features which are basically whatever the client gets from the facility supplier. It is usually hard for patients to assess the medical quality which they are getting [29]. The medical consequence of a specific process is not instantly visible in several conditions [30]. Furthermore, views of a patient’s medical treatment can be assessed during healthcare delivery. During the continuation of care, patient perceptions vary from time to time [29].

The abilities of a service provider [31], choosing an appropriate diagnostic instrument, and suitable timing for the treatment are the essential features of medical quality (MQ) [32]. Technical aspects of healthcare are categorized as the choice of client from illness or inability, doctor and paramedical staff care, and healthiness level after being admitted to the hospital [8]. Unintentional re-admissions would be reflected in the effect of medical results not being suitable sometimes. One approach to advance medical quality is to create guidelines concentrating on medical care quality with the aim of refining the consequences of the medical processes [33].

### 2.3. Administrative Quality

Administrative Quality (AQ) means the way in which services are provided to the patients. It contains formulating the experience of the patient in the healthcare institution to treat efficiently. Features of administrative quality involved the personalization and client-service provider relationship, provision of medicine and foodstuff to the patient, the effectiveness of admission and discharge procedure, and appropriateness payment invoices [22]. Usually, it is comfortable for the clients to evaluate the degree of administrative quality the same way as for medical quality [34], such as patients might not be sure of the quality of their operation. However, they feel they were given better care and treatment or treated inadequately by the physicians and nursing staff [35].

Poor administrative quality can also lead to reduced medical quality [14]. In the MBNQA Healthcare Criterion, important considerations are given to the patient’s care including a strategy of support and service procedures, the ways of service delivery, and the participation of the patients in order to create necessary modifications or enhancements [10]. It focusses more on how the healthcare personnel (nurses, physicians, administration, etc.) developed the abilities to provide desired patient-centered care and improve the medical and administrative quality of this care [36].

### 2.4. Patient Satisfaction

There is a substantial quantity of literature on what factors can affect the level of Patient Satisfaction (PS). Hussain, et al. [37] referred to client satisfaction as “the result of satisfaction with a series of transactions occurring during the service process”. The quality and efficiency of services provided to the clients determined overall satisfaction [3]. Since client satisfaction is strongly linked to loyalty, it is possible that the client felt more confidence and in return, he/she advised others to use these services. Prior research demonstrated that it is very important for the healthcare organizations to respect patients’ perceptions and feedback regarding the services they received which would ultimately help the organizations to improve service quality [6,22].

Nowadays healthcare regulatory bodies believed patient satisfaction to be an important predictor of healthcare quality [37]. According to Hussain et al. [37], patient satisfaction must integrate several care aspects including technical, personal, communal, and ethical. Since healthcare organizations try to provide the best services to their clients by directly contacting them, the desired quality level can be varied and in return, the relationship between patients and the service provider is affected [36].

### 2.5. Leadership and Patient Satisfaction

Prior research has shown that managers with an effective leadership style positively affect staff behavior and patient satisfaction [14,38,39]. Effective leadership style helps the leaders to acts as a spiritual mentor to attain desired goals not only in terms of patient satisfaction, but also in all other aspects of the hospital environment [40]. According to Bahadori et al. [4], task oriented leadership is more influential on patient satisfaction than relationship oriented leadership. According to Asif et al. [14] transformational leadership style in the field of healthcare in general, and nursing in particular, has received more attention, because it improves job satisfaction, facilitates change process and increases organizational commitment and welfare among employees which ultimately leads to enhanced patient satisfaction.

### 2.6. Leadership and Administrative Quality

In the context of managerial influence on healthcare institutional quality, West [41] argues that participative leadership (PL) involves making strong efforts to develop a healthy work environment that encourages employees to participate in the decision-making process and increases their communication level. Similarly, support from top leadership plays an important role in service delivery and strengthens the relationship between administrative staff and patients [42]. Guillén and González [43] observed effective leadership as an indispensable prerequisite for the implementation of every persistent and progressive setup. Schaefer et al. [10] proved that effective leadership has a significant causative effect on various factors of the Baldrige system e.g., procedure management, service provision, design, and introduction of any healthcare delivery scheme.

### 2.7. Leadership and Medical Quality

To attain better medical quality, Giangrande [44] argued that healthcare organizations are needed to develop a management culture with a vision to utilized resources aiming to improve patient-centered care and satisfaction. Asif et al. [14] explored the association between leadership and organization systems and their influences on outcomes for healthcare organizations. Their research exposed that service quality can be improved in healthcare organizations and leadership positively affects staff satisfaction, which ultimately enhances patient satisfaction. Shortell, et al. [45] found a significant link between the dealing of the service provider and patient assessed technical quality. In their study, the factor dealings of the service provider contained nurse and doctor’s leadership style and staffs’ influential capability aiming to attain desired organizational goals. These types of research provided a significant association between leadership and medical quality.

### 2.8. Administrative Quality and Patient Satisfaction

Schaefer et al. [10] found managerial procedure as an influential element in patient satisfaction (PS). Hoque et al. [29] established an instrument to assess in what way the features of administrative quality (AQ) regarding clinics’ experience influence patient satisfaction (PS). They noticed that improving several healthcare services (efficient facility provision, information accessibility, supportive behavior, and effective staff-patient interaction) may lead to a higher satisfaction level among patients. Meyer [28] explored a causative association between administrative procedure and client satisfaction, though the clients were assured to involve other participants consistent with the MBNQA Criterion. DE Man et al. [46] discovered a significant relation between service quality delivered to the patients and their satisfaction by using the multidimensional service quality (SERVQUAL) instrument. In another research conducted in the emergency department, authors found staff-patient interaction as the most dominant factor of patient satisfaction where patients’ feel more gratification with the services they are delivered [47].

### 2.9. Medical Quality and Patient Satisfaction

Hussain et al. [37] discovered that patient satisfaction is highly linked to medical outcomes. In general, patients are more satisfied if they experienced better health service delivery with greater care. On the other hand, Asif et al. [14] revealed that patient-centered medical outcomes are as significant as nursing and staff care. They further proved that with the help of effective leadership, these outcomes can be improved which resulted in greater patient satisfaction. Meesala and Paul [48] established a strong relationship between technical (medical) quality and patient satisfaction (PS). They conceptually argued that medical quality reacts to patient satisfaction differently, and it depends on the organizational situation in which it is delivered.

**Hypothesis** **1:** *There will be a significant and positive relationship between participative leadership and administrative quality.*

**Hypothesis** **2:** *There will be a significant and positive relationship between participative leadership and medical quality.*

**Hypothesis** **3:** *There will be a significant and positive relationship between administrative quality and patient satisfaction.*

**Hypothesis** **4:** *There will be a significant and positive relationship between medical quality and patient satisfaction.*

**Hypothesis** **5:** *Administrative quality will mediate the relationship between participatory leadership and patient satisfaction.*

**Hypothesis** **6:** *Medical quality will mediate the relationship between participatory leadership and patient satisfaction.*

## 3. Materials and Methods

### 3.1. Sample and Design

We established a survey questionnaire according to the MBNQA Criteria and all items were rated by using a 5-point Likert scale where 5 is for “strongly agree” and 1 is for “strongly disagree”. The survey questionnaire was sent via mail to the head of hospital management and quality assurance department. We selected 145 public sector hospitals in Pakistan to obtain the required data. These hospitals are situated in the Punjab province including 23 teaching hospitals, 34 district headquarter hospitals (DHQ), and 88 tehsil headquarter hospitals (THQ). All these hospitals have more than 50 beds [49]. A total number of 129 surveys were received for this study. Out of 129 surveys, 6 surveys were removed because of missing values, and the remaining 123 responses were used to evaluate the study with a percentage of 84.83 (true responses used in the study). Additionally, a non-response bias test was conducted to assure the significant differences between completed surveys and the institution size. Values of the χ^2^ difference test for institution size (χ^2^ = 4.23, df = 6, *p* = 0.71) and type of institution (χ^2^ = 3.64, df = 3, *p* = 0.22) were insignificant [28]. According to Hair [50], the sample size for a structural model should be based on 5 to 10 respondents for each item. This study contained 12 questions with total responses of 123.

For testing the hypothesized model, we used confirmatory factor analysis (CFA) and structural equation modeling (SEM) tests. We also developed a measurement model which included the links between studied factors and their items. We used a three-stage strategy of SEM as recommended by Qing et al. [26]. The study first performed a measurement model to evaluate component scores for all items. Secondly, CFA was utilized to check the discriminant validity, and thirdly, the SEM technique was adopted to evaluate the causal model [51]. These steps were taken to confirm different types of validities and reliabilities of the causal model.

### 3.2. Measures

All measures are based on Baldrige Health Care Criteria where participative leadership (PL) was measured using a 3-items scale with Cronbach’s alpha of 0.81. The example items for participative leadership included “Our senior executives are involved in quality activities” and “Our senior executives focus on improving patient care”. Administrative quality (AQ) has 3 items, and its Cronbach’s alpha value was 0.80. The sample questions for administrative quality were “Patient preferences are analyzed when designing new and revised patient services” and “Our service design is continuously improved”. Medical quality (MQ) contained 3 items. The α coefficient was 0.75 for medical quality scale. The sample question for medical quality was “Clinical outcomes measured internally for specific diagnosis” and “Clinical outcomes measured externally for specific diagnosis”. Patient satisfaction (PS) consisted of 3 items with sample questions including “Overall satisfaction of patients” and “Number of patients who return for future visits”. The α reliability for patient satisfaction was 0.79.

## 4. Results

### 4.1. Descriptive Statistics

Descriptive statistics including mean, standard deviation, and correlation among all constructs are demonstrated in Table 1. It can be seen that correlation values between participative leadership and administrative quality (*r* = 0.41; *p* < 0.01), participative leadership and medical quality (*r* = 0.37; *p* < 0.01), participative leadership and patient satisfaction (*r* = 0.39; *p* < 0.01), administrative quality and patient satisfaction (*r* = 0.38; *p* < 0.01), and medical quality and patient satisfaction (*r* = 0.31; *p* < 0.01) are statistically significant and positive. The values of mean and standard deviation for participative leadership (3.97, 0.79), administrative quality (4.13, 0.93), medical quality (3.41, 0.78), and patient satisfaction (3.11, 0.63) are noted. Bold values in Table 1 showed the discriminant validity of the variables. The discriminant validity for participative leadership, administrative quality, medical quality, and patient satisfaction are 0.81, 0.82, 0.79, and 0.83, respectively. All these values are the square root of average variance extracted (AVE) and are larger than the values of construct’s inter-correlations [14].

### 4.2. Measurement Model

Our study consisted of four variables including participative leadership (PL), administrative quality (AQ), medical quality (MQ), and patient satisfaction (PS). Cronbach’s α, factor loadings, standard errors, t-statistics, AVE, and composite reliabilities (CR) for these variables are demonstrated in Table 2. αs for participative leadership, administrative quality, medical quality, and patient satisfaction are 0.81, 0.80, 0.75, and 0.79, respectively. Cronbach’s α is considered the most important technique to test the internal consistency. The accepted criteria for α is 0.70 [52]. The range of factor loadings for participative leadership, administrative quality, medical quality, and patient satisfaction is 0.80–0.89, 0.83–0.89, 0.76–0.81, and 0.76–0.85, respectively. The accepted criteria for factor loading is 0.50 [50,53]. The values of t-statistics for participative leadership, administrative, medical quality, and patient satisfaction are 15.69–16.96, 16.60–17.45, 15.80–15.88 and 14.90–16.04, respectively, and are higher than the minimum accepted criterion value of 1.96 [14,26]. We measured the convergent validity of all variable through the values of AVE. In our study the AVE value for participative leadership (0.65), administrative quality (0.68), medical quality (0.63) and patient satisfaction (0.69) are above than the minimum criterion value of 0.50 [54]. Moreover, the value of CR should be greater than 0.60 as suggested by Bagozzi and Yi [55] while in our study, we noted the CR values for participative leadership (0.87), administrative quality (0.84), medical quality (0.83), and patient satisfaction (0.80).

### 4.3. Confirmatory Factor Analysis

Before testing study hypotheses, we measured the discriminant validity of our model using CFA. CFA is the most important and widely accepted tool that is used in doing mediation analysis [26]. Table 3 demonstrated the results of CFA where the 4-factor model, which is actually a baseline model in our study, is compared with 5 alternative models (three 3-factor models, one 2-factor model, and one 1-factor model). In the first 3-factor model, we combined participative leadership and administrative quality into a single factor, in the second 3-factor model, we combined participative leadership and medical quality and in the third 3-factor model, we combined administrative quality and medical quality into a single factor. In the 2-factor model, we combined participative leadership, administrative quality, and medical quality into a single factor. In the 1-factor model, all constructs of the study are loaded into a single factor. We used χ^2^, χ^2^/df, CFI, IFI, TLI, and RMSEA as the fit indices during the CFA analysis where χ^2^/df should be <3 [14], values of CFI, IFI and TLI should be ≥0.90 [56] and RMSEA should be <0.06 [57]. Our findings showed that the 4-factor model is the best-fitted model since it has the most suitable values. These values included χ^2^ = 590.51, χ^2^/df = 1.44, CFI = 0.97, IFI = 0.96, TLI = 0.97 and RMSEA = 0.031.

### 4.4. Hypotheses Testing

We used SEM to test the hypotheses with the help of AMOS 25.0 (SPSS Inc., Chicago, IL, USA) [58]. In the present study, hypothesis 1 was ‘there will be a significant and positive relationship between participative leadership and administrative quality’. With the help of Table 1 and Table 4, we found hypothesis 1 significant and positive because the values of correlation (r = 0.41; *p* < 0.01) and regression (β = 0.46; t = 12.43; *p* < 0.01) provide supportive evidence. Hypothesis 2 of the present research was ‘there will be a significant and positive relationship between participative leadership and medical quality’. The coefficients of correlation (r = 0.37; *p* < 0.01, see Table 1) and regression (β = 0.32; t = 8.42; *p* < 0.01, see Table 4) provide enough support to hypothesis 2. Hypothesis 3 of the present research was ‘there will be a significant and positive relationship between administrative quality and patient satisfaction’. The results of Table 1 and Table 4 support hypothesis 3, since the correlation (r = 0.38; *p* < 0.01) and beta coefficient (β = 0.39; t = 9.51; *p* < 0.01) between administrative quality and patient satisfaction were significant and positive. Hypothesis 4 of the present research was ‘there will be a significant and positive relationship between medical quality and patient satisfaction’. We found supportive evidence from Table 1 and Table 4 with the correlation (r = 0.31; *p* < 0.01) and regression estimates (β = 0.26; t = 6.84; *p* < 0.01). We also measured a 95 % confidence interval (CI) for Table 4.

**Figure 1:**
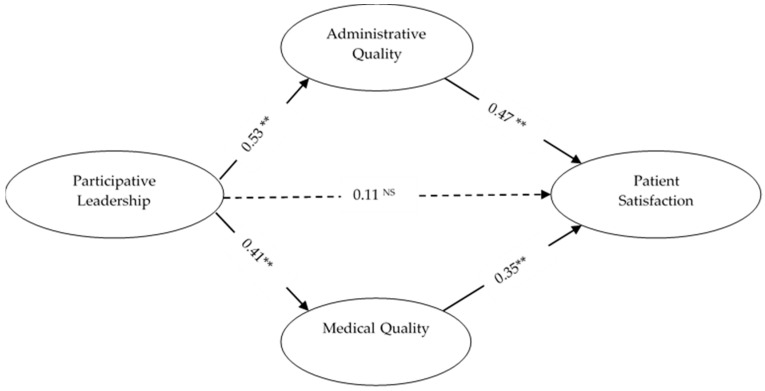
Mediation effects using SEM. ** *p* < 0.01, NS: Non significant

Figure 1 indicated the mediating relationships for hypotheses 5 and 6. As exhibited in Figure 1, we found path estimates from participative leadership to administrative quality (β = 0.53; *p* < 0.01), participative leadership to medical quality (β = 0.41; *p* < 0.01), administrative quality to patient satisfaction (β = 0.47; *p* < 0.01) and medical quality to patient satisfaction (β = 0.35; *p* < 0.01) significant and positive. On the other hand, the path estimate from participative leadership to patient satisfaction (β = 0.11; *p* > 0.05; not supported) was insignificant. These results indicated full mediation and support hypotheses 5 and 6. In addition, we estimated the indirect effects of mediating variables as recommended by Preacher and Hayes [59]. We performed bootstrapping at a 95% confidence interval (CI) with 5,000 boot samples and calculated the confidence intervals (CI) of the lower (LLCI) and upper bounds (ULCI) as well as *z* values. The bootstrapping analysis for indirect effects are exhibited in Table 5, where we found significant mediating effects of administrative quality on PL-PS relationship (β = 0.25; z = 4.63 and *p* < 0.01) and medical quality on PL-PS relationship (β = 0.14; z = 2.75 and *p* < 0.01). These results provide additional support for hypotheses 5 and 6.

## 5. Discussion and Implication

The present research used CFA and SEM techniques to investigate and validate the structural relationships proposed in the MBNQA criteria. These findings help MBNQA criteria to advance its structural model and enable the health institutions to decide how to achieve organizational goals and how to enhance patient satisfaction with limited resources [11]. Under the Baldrige Healthcare model, the MBNQA criteria have become the most accepted performance standard especially in the health sector [10]. Findings of the present study offer empirical evidence that the causal relationships using the structural model (under MBNQA criteria) provide greater insights to understand how the performance of healthcare institution can be defined and enhanced. In this regard, the purpose of this research was to investigate the impact of participative leadership on patient satisfaction through the intervening influences of administrative quality and medical quality. Since leadership played a significant role in enhancing clients’ satisfaction [4], no research has been done to evaluate the influence of participative leadership on patient satisfaction through administrative quality and medical quality.

We have gained some interesting insights from this study. First, the result in Table 4 revealed that two paths from participative leadership to administrative quality and participative leadership to medical quality are significant. This outcome proves the previous studies that leadership models help to improve the quality control system [9,60]. Moreover, these results emphasize that participative leadership is strongly connected to administrative quality as compared to medical quality. Second, it is essential to note that the relationship between administrative quality and patient satisfaction (β = 0.47) is significantly higher than the relationship between medical quality and patient satisfaction (β = 0.47). We have accounted for the relative precision of path evaluation and the 95% confidence interval of both are given in Table 4. Third, it can be seen from Figure 1 that the direct path from participative leadership to patient satisfaction (β = 0.11) disappeared in the presence of administrative quality and medical quality. This showed that healthcare leadership has much more of an impact on the implementation of policies, process design and to improve the quality in the hospital in order to enhance patient satisfaction [4]. The significant relationship here recognized that participative leadership seriously aimed to play its role to improve service quality in the health sector. This can be achieved if hospital top management took some serious actions in terms of the medical staff’s care with a well-trained physician team and used the latest diagnostic methods to provide medical care under the compliance and standard care patterns of the hospitals [6,14]. Fourth, the significant and positive indirect effect of participative leadership through administrative (β = 0.25; *p* < 0.01) and medical (β = 0.14; *p* < 0.01) quality in improving healthcare quality management system indicated the necessity of adopting an effective leadership style in order to improve hospital services’ quality, which ultimately leads to improve patient satisfaction. It further enables the top management to focus on improving the process and service experience that patients can judge and evaluate [61].

In a healthcare service delivery system, medical quality (MQ) should be on the top priority level of hospital managers, patients, and doctors. Medical quality is an essential component of the health care system because it can have more influential medical strategies based on training, expert database, and scientific research [6]. Prior research has found that patients have fewer resources and opportunities to evaluate the medical quality facilities provided by the hospital, while administrative quality can be easily assessed by patients and their family because they interact with administrative areas more directly [33,62,63]. Top management need to find out how patients assess the administrative quality of their facilities and then provide them better ways to evaluate medical quality in the same way as administrative quality. This will also increase patient satisfaction.

Finally, the top-level management in healthcare institutions needs to make sure their medical quality is equally important to the administrative quality of their facilities. It is understood that both medical quality and administrative quality are of equal importance [9], meaning in the context of Pakistan, based on our results, medical quality needs to be improved.

## 6. Limitation of the Study

First, our study only focused on two types of quality as the mediators (administrative quality and medical quality), meaning future research should use other qualities as intervening variables, such as information quality to comprehensively understand the influence of participative leadership on healthcare outcomes. Second, our study sample consisted of government hospitals, so future studies should incorporate both public and private hospitals with a wider area. Finally, care should be taken in generalizing the results of this study. While some argue that service quality in the healthcare industry addresses issues relevant to healthcare delivery (complexity, co-production, and intangibility) that have been supported to be generalizable to other service contexts [64,65], we would also be mindful of the nature of service quality in healthcare organizations. We expect that the findings of this study can be generalizable to industries with service expectations similar to those of the healthcare industry, where there is a significant knowledge gap and information asymmetry between the service provider and the customer (e.g., auto repair, consulting, or law firms).

## 7. Conclusion

In this research, we have presented a model structural model using MNBQA criteria. We examined the effect of participative leadership and two types of qualities (administrative and medical) on patient satisfaction. The role of effective leadership in creating a healthy system in order to deliver quality services is crucial and needs the technical abilities to control both administrative and medical quality. It is very important to include all features of administrative quality including designing, hiring, training to staff, behavior, and rewarding the best employees while designing a healthcare delivery system. Since healthcare institutions have tried to provide better services to their clients, they need to develop several measures that would enable patients to feel more satisfaction. Our research can be helpful to understand the patients’ requirements and capacity to control and forecast the organization’s outcomes according to customer needs. The empirical results of this research emphasize that a service delivery system should focus on both administrative quality and medical quality of facilities to maximize patient satisfaction.

## Figures and Tables

**Table 1:** Descriptive statistics, correlations and discriminant validity.

Factor	Mean	SD	Correlations			
			1	2	3	4
1. PL	3.97	0.79	**(0.81)**			
2. AQ	4.13	0.93	0.41 **	**(0.82)**		
3. MQ	3.41	0.78	0.37 **	0.46 **	**(0.79)**	
4. PS	3.11	0.63	0.39 **	0.38 **	0.31 **	**(0.83)**

** *p* < 0.01. Square root of AVE (bold values) are shown in parenthesis demonstrating discriminant validity. SD: standard deviation; PL: participative leadership; AQ: administrative quality; MQ: medical quality; PS: patient satisfaction; AVE: average variance extracted.

**Table 2:** Overall measurement model.

Factor		Factor Loading	SE	t	AVE	α	Composite Reliability (CR)
Participative leadership (PL)					0.65	0.81	0.87
PL1	Our senior executives are involved in quality activities	0.89	-	-			
PL2	Our senior executives focus on improving patient care	0.83	0.049	16.94			
PL3	Our senior executives are accessible to patients	0.80	0.051	15.69			
Administrative quality (AQ)					0.68	0.80	0.84
AQ1	Patient preferences are analyzed when designing new and revised patient services	0.87	-	-			
AQ2	Our service design is continuously improved	0.89	0.051	17.45			
AQ3	Easing customer access to information that they need (degree of emphasis)	0.83	0.050	16.60			
Medical quality (MQ)					0.63	0.75	0.83
MQ1	Patient unplanned readmissions	0.76	-	-			
MQ2	Clinical outcomes measured internally (relative to competitors)	0.81	0.051	15.88			
MQ3	Clinical outcomes measured externally (relative to competitors)	0.79	0.050	15.80			
Patient satisfaction (PS)					0.69	0.79	0.80
PS1	Overall patient satisfaction (relative to competitors)	0.82	-	-			
PS2	Overall satisfaction of patients	0.76	0.051	14.90			
PS3	Number of patients who return for future visits	0.85	0.053	16.04			

Note: SE: standard error; AVE: average variance extracted.

**Table 3:** Confirmatory Factor Analysis (CFA).

Model	χ^2^	Df	χ^2^/df	Δχ^2^	Δdf	CFI	IFI	TLI	RMSEA
4-Factor model (Baseline)	590.51	409	1.44	-	-	0.97	0.96	0.97	0.031
3-Factor model (PL + AQ combined)	643.13	411	1.56	52.62	2	0.89	0.90	0.89	0.052
3-Factor model (PL + MQ combined)	624.27	411	1.52	33.76	2	0.91	0.90	0.91	0.047
3-Factor model (AQ + MQ combined)	701.49	414	1.69	110.98	5	0.87	0.85	0.83	0.072
2-Factor model (PL + AQ + MQ combined)	835.77	416	2.01	245.26	7	0.81	0.79	0.80	0.078
1-Factor model	923.07	415	2.22	332.56	6	0.78	0.78	0.79	0.096

Note: PL: participative leadership; AQ: administrative quality; MQ: medical quality; df: degree of freedom; CFI: comparative fit index; IFI: incremental fit index; TLI: Tucker-Lewis index; RMSEA: root-mean-square error of approximation.

**Table 4:** β estimates for testing hypotheses 1–4.

Hypotheses	Path	β Estimate	SE	T	95% Confidence Interval	*p*-Value	Sig.
H1	PL 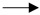 AQ	0.46	0.037	12.43	(0.437, 0.551)	<0.01	(**)
H2	PL 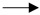 MQ	0.32	0.038	8.42	(0.179, 0.421)	<0.01	(**)
H3	AQ 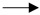 PS	0.39	0.041	9.51	(0.328, 0.493)	<0.01	(**)
H4	MQ 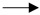 PS	0.26	0.038	6.84	(0.163, 0.339)	<0.01	(**)

Note: PL: participative leadership; AQ: administrative quality; MQ: medical quality; PS: patient satisfaction.

**Table 5:** Bootstrapping analysis for indirect effects.

Mediating Effects	Boot Indirect Effect	Boot SE	Boot z	Sig.	95% Confidence Interval	
					LLCI	ULCI
PL 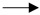 AQ 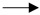 PS	0.25	0.054	4.63	(**)	0.13	0.35
PL 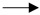 MQ 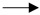 PS	0.14	0.051	2.75	(**)	0.18	0.31

Note: PL: participative leadership; AQ: administrative quality; MQ: medical quality; LLCI: lower limit confidence interval; ULCI: upper limit confidence interval. ** *p* < 0.01.
